# Rituximab as First‐Line Compared to Escalation Immunotherapy Is Associated With Lower Disability Accumulation in Aquaporin‐4‐IgG‐Positive Neuromyelitis Optica Spectrum Disorder: A Multicenter Cohort Study From Germany and the United Kingdom

**DOI:** 10.1111/ene.70243

**Published:** 2025-06-10

**Authors:** Daniel Engels, Chiara Rocchi, Mirasol Forcadela, Pakeeran Siriratnam, Marius Ringelstein, Martin W. Hümmert, Katrin Giglhuber, Achim Berthele, Corinna Trebst, Orhan Aktas, Joachim Havla, Saif Huda, Tania Kümpfel

**Affiliations:** ^1^ Institute of Clinical Neuroimmunology and Biomedical Center (BMC), LMU University Hospital, Faculty of Medicine Ludwig‐Maximilians‐University Munich Munich Germany; ^2^ Department of Neurology Walton Centre NHS Foundation Trust Liverpool UK; ^3^ School of Translational Medicine Monash University Clayton Victoria Australia; ^4^ Department of Neurology Medical Faculty and University Hospital Düsseldorf, Heinrich Heine University Düsseldorf Duesseldorf Germany; ^5^ Department of Neurology, Center for Neurology and Neuropsychiatry LVR‐Klinikum, Heinrich Heine University Düsseldorf Düsseldorf Germany; ^6^ Department of Neurology Hannover Medical School Hannover Germany; ^7^ Department of Neurology, School of Medicine and Health Technical University Munich, Klinikum Rechts der Isar Munich Germany; ^8^ University of Liverpool Liverpool UK; ^9^ Walton Centre NHS Foundation Trust Liverpool UK

**Keywords:** azathioprine, immunosuppression, mycophenolate mofetil, neuromyelitis optica spectrum disorder, rituximab

## Abstract

**Background:**

Aquaporin‐4‐IgG‐positive neuromyelitis optica spectrum disorder (AQP4‐IgG+ NMOSD) can cause significant disability after a single attack. Long‐term immunotherapy reduces disability accumulation, but the choice of first‐line therapy varies. In the United Kingdom, rituximab is typically used as escalation therapy after conventional immunosuppressants fail, while in Germany, it is widely used as first‐line treatment.

**Methods:**

We compared attack risk and disability outcomes based on the Expanded Disability Status Scale (EDSS) in AQP4‐IgG+ NMOSD patients treated with rituximab as first‐line versus escalation therapy. Furthermore, attack suppression and risk factors for attacks in individuals who received treatment with azathioprine and mycophenolate mofetil (with/without escalating to rituximab) were analyzed.

**Results:**

The risk of attack was lower in individuals who received rituximab as first‐line therapy (*n* = 52) compared to escalation therapy (*n* = 81, HR = 0.45, 95% CI = 0.30–0.67). Once escalated to rituximab, there was no altered risk of attack between first‐line rituximab and escalation therapy (HR = 1.15, 95% CI = 0.64–2.08). Rituximab as first‐line compared to escalation therapy was associated with lower EDSS scores at therapy start (3.0 vs. 6.0, *p* = 1.10 × 10^−3^). In patients who remained on azathioprine or mycophenolate mofetil (*n* = 45), age < 50 years and treatment with azathioprine were identified as risk factors for attacks.

**Conclusions:**

Rituximab as a first‐line therapy shows significant reduction in disability accumulation compared to escalation treatments. However, a subgroup of patients with AQP4‐IgG+ NMOSD may still respond well to conventional immunosuppression—specifically older patients treated with mycophenolate mofetil.

## Graphical abstract

In this study, we compared clinical outcomes in AQP4‐IgG+ NMOSD patients treated with rituximab as first‐line versus escalation immunotherapy. While the risk of attacks was similar once rituximab was initiated in both groups, patients receiving rituximab as escalation therapy had higher EDSS scores at treatment start and at the end of follow‐up. Among those on conventional immunosuppressants (e.g., before escalation to rituximab), younger age (< 50 years) and treatment with azathioprine (vs. mycophenolate mofetil) were associated with a higher risk of attacks.

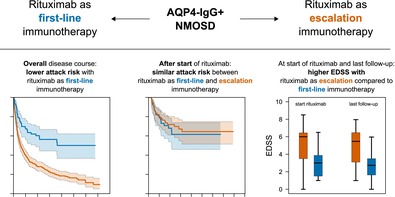

## 1 Introduction

Aquaporin‐4‐IgG‐positive neuromyelitis optica spectrum disorder (AQP4‐IgG+ NMOSD) is an autoimmune disease of the central nervous system that primarily manifests with optic neuritis and longitudinally extensive transverse myelitis [1]. AQP4‐IgG+ NMOSD is classically relapsing, and a single attack may be associated with severe disability [2]. Therefore, effective long‐term immunotherapy to prevent attacks is an essential component of AQP4‐IgG+ NMOSD management.

Several immunotherapies with a broad spectrum of mechanisms of action have been used to treat AQP4‐IgG+ NMOSD during the past decades. These included conventional immunosuppressants such as azathioprine (AZA), methotrexate (MTX), and mycophenolate mofetil (MMF), (oral) steroids, or monoclonal antibodies such as rituximab and tocilizumab [3, 4, 5, 6, 7, 8]. Treatment with these medications is off‐label and rituximab was only approved in Japan after a randomized controlled trial [7]. Recently several approved monoclonal antibodies (inebilizumab, satralizumab, eculizumab, and ravulizumab) have become available for AQP4‐IgG+ NMOSD treatment [9, 10, 11, 12]. According to recent recommendations, these monoclonal antibodies including rituximab should be considered as first‐line therapy for AQP4‐IgG+ NMOSD [13, 14]. However, due to differences in health care policies as well as socioeconomic disparities in health care, free choice of therapy is not available in every country.

There is evidence that rituximab is superior to conventional immunosuppression in reducing AQP4‐IgG+ NMOSD attack rate and that early initiation of rituximab may prevent long‐term disability [15, 16]. Thus, patients in whom conventional immunosuppression is not sufficient to suppress attacks or who are severely affected by a first attack with residual disability would presumably benefit from early therapy with rituximab. Moreover, rituximab was already recommended as a first‐line AQP4‐IgG+ NMOSD treatment by the European Federation of Neurological Societies and by the German Neuromyelitis Optica Study Group (NEMOS) more than 10 years ago [17, 18].

We hypothesized that different access schemes to rituximab would influence the outcome of AQP4‐IgG+ NMOSD therapy. Thus, we compared treatment efficacy (risk of attack and disability worsening) between rituximab as first‐line and escalation treatment in a German and United Kingdom AQP4‐IgG+ NMOSD cohort and assessed risk factors for attacks. Furthermore, we analyzed risk factors for attacks during azathioprine or mycophenolate mofetil treatment episodes (before escalating to rituximab and with ongoing azathioprine or mycophenolate mofetil treatment).

## 2 Methods

### 2.1 Study Cohort and Data Acquisition

We performed a retrospective observational longitudinal study. Our study cohort comprised patients with AQP4‐IgG+ NMOSD from specialized neuroimmunology centers in the United Kingdom (Liverpool) and Germany (Duesseldorf, Hannover, Munich). The diagnosis was made according to the 2015 diagnostic criteria of the International Panel for NMO Diagnosis (IPND) [1]. We included patients who received at least one cycle of rituximab during their disease either as first‐line (first long‐term immunotherapy after diagnosis was made) or as escalation therapy. We also incorporated subjects treated with (ongoing) azathioprine or mycophenolate mofetil, who had not switched to rituximab. The (retrospective) observation period was from 1997 to 2023. Demographic data (year of birth, sex), date of disease manifestation and initial symptom, course of immunotherapies (start and end date), attacks, and Expanded Disability Status Scale (EDSS) scores were retrospectively collected by reviewing medical records and partially also extracted from the NEMOS registry (prospective follow‐up of patients) in German patients and from a local database for United Kingdom patients. Patients with incomplete data were excluded from the respective analysis.

### 2.2 Outcomes

We analyzed attacks that occurred under therapy (at least 6 months after start of therapy) and estimated the risk of attack with a time‐to‐event analysis. We only included rituximab, azathioprine, and mycophenolate mofetil treatment episodes in this analysis. Clinical parameters for the risk of attacks were evaluated. For the analysis of attack risk over the entire disease course, we included attacks that occurred from 6 months after the initiation of the first immunotherapy up to the time of the last follow‐up.

To evaluate disability progression, we examined EDSS scores recorded 30 days post‐attack. We gathered EDSS scores from the initial follow‐up through the last follow‐up or, if rituximab therapy was discontinued, up to that point. Only EDSS scores collected over a period exceeding 6 months were included in the analysis. Disability/EDSS progression was defined as an increase in the EDSS score by 1.5 points for a baseline score of 0, an increase by 1.0 point for a baseline score between 1.5 and 5.5, or an increase by 0.5 points for a baseline score of 6.0 or higher [19]. To estimate the level of disability at the start of rituximab, we calculated the median EDSS score from all EDSS scores recorded within 6 months before and 6 months after the initiation of rituximab therapy.

### 2.3 Ethics

All patients gave their written consent for contributing to retrospective data analyses. Ethical approval for Liverpool was granted by the London‐Hampstead Research Ethics Committee (15/LO/1433). All patients from German centers were included in the NEMOS registry, and ethical approval was granted by each local ethics committee.

### 2.4 Data Analysis

Central tendencies and dispersions for quantitative variables were expressed as median (Md) and interquartile range (IQR, 25th to 75th percentile). We performed the Mann–Whitney *U* test to test whether there is a difference between the central tendencies of a non‐parametric variable between two groups. The relationship between categorical variables was estimated by the chi‐squared test or Fisher's exact test (if the absolute number of cases per contingency table field was below 5). For time‐to‐event analyses, we calculated the time between the start of immunotherapy and the attack, the time between attacks, and the time between the last attack and the stop of the respective immunotherapy (recurring events). To assess the influence of independent variables on the risk of attack after the initiation of therapy, we calculated mixed‐effects Cox proportional hazards models with subject ID as a random effect and reported hazard ratios (HR) with 95% confidence intervals (95% CI), and *p* values (for H_0_: HR = 1). Data analysis was performed with a script written in Python (3.11.5) and R (4.5.0).

## 3 Results

### 3.1 Study Cohort

Our study cohort consisted of 178 AQP4‐IgG+ NMOSD patients (Germany: *n* = 95, United Kingdom: *n* = 83) who received rituximab as first‐line (*n* = 52, all Germany) or escalation therapy (*n* = 81, Germany: *n* = 35) or with ongoing treatment with azathioprine or mycophenolate mofetil (*n* = 45, Germany: *n* = 8). In the German subcohort, the majority of patients received rituximab as first‐line therapy, whereas in the United Kingdom, no patient received rituximab as first‐line therapy. In contrast, azathioprine and mycophenolate mofetil were predominantly used as first‐line treatments in the United Kingdom, and more patients with ongoing treatment with azathioprine or mycophenolate mofetil were identified in the United Kingdom subcohort. Except for a slightly longer observation time of patients in the United Kingdom subcohort, there were no differences in age at diagnosis, proportion of females, or disease phenotype at onset between the United Kingdom and German subcohorts. The median observation time (including retrospective chart analysis) was 8.34 years (IQR = 5.00–11.61 years). The median age at diagnosis was 48 years (IQR = 37.75–57 years). The sex ratio was in favor of women (proportion of females: 0.85). Myelitis was the most frequent initial symptom, followed by optic neuritis (Table 1).

**TABLE 1:** Demography of the study cohort.

		Germany	United Kingdom	*p*
Number of patients		95	83	—
Rituximab first‐line/rituximab escalation/ongoing azathioprine or mycophenolate mofetil (*n*)		52/35/8	0/46/37	—
Observation time, median years (IQR)		7.52 (4.43–11.01)	9.19 (5.76–12.05)	0.03
Age at diagnosis, median years (IQR)		49 (38–59)	46 (36.5–56)	0.37
Females, proportion		0.85	0.86	> 0.99
Myelitis/ON/other phenotype at onset, proportion		0.46/0.35/0.19	0.49/0.36/0.14	0.73
Number of first‐line therapies (proportion)	Rituximab	52 (0.55)	0 (0.00)	—
	Azathioprine	27 (0.28)	54 (0.65)	—
	Mycophenolate mofetil	2 (0.02)	25 (0.30)	—
	Methotrexate	2 (0.02)	0 (0.00)	—
	Intravenous immunoglobulins	1 (0.01)	0 (0.00)	—
	Oral steroids	0 (0.00)	2 (0.02)	—
	Other	11 (0.12)	2 (0.02)	—

*Note:* AQP4‐IgG+ NMOSD patients treated with rituximab as first‐line or escalation therapy or with ongoing conventional immunosuppression with azathioprine or mycophenolate mofetil in Germany and the United Kingdom. *p* values result from Mann–Whitney *U* test (observation time, age at diagnosis) or chi‐squared test (sex distribution, proportion of disease phenotype at onset; IVIG: intravenous immunoglobulins, IQR:, interquartile range).

### 3.2 Risk of Attack

**FIGURE 1:**
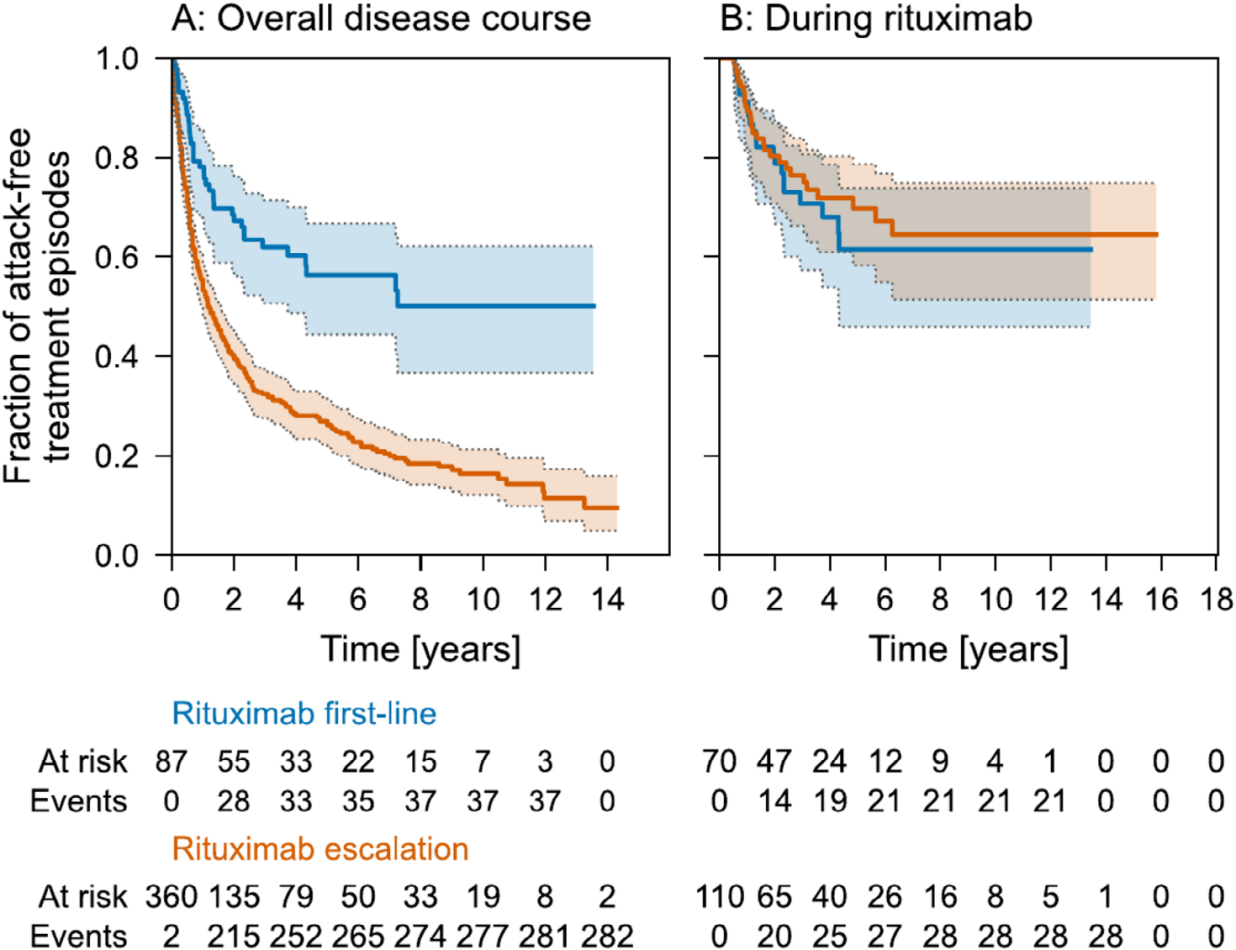
Risk of attacks. Time‐to‐event (attack) analysis with recurring events of AQP4‐IgG+ NMOSD patients who received rituximab first‐line (blue, *n* = 52 patients) or as escalation treatment (orange, *n* = 81 patients). Attacks over the entire disease course since the start of first immunotherapy (A) and only during rituximab treatment episodes (B) were considered. Numbers at risk and event counts correspond to treatment episodes.

**FIGURE 2:**
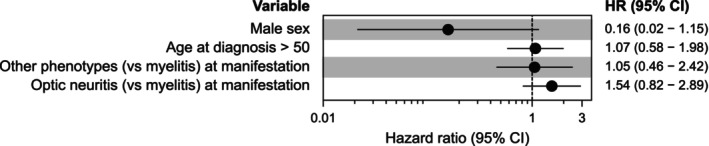
Risk factors for attacks under rituximab (administered as first‐line or escalation therapy). Multivariable mixed‐effects Cox proportional hazards model based on recurring time‐to‐event analysis with patient as a random effect. Dots represent hazard ratios, horizontal lines represent 95% confidence intervals. Lower hazard ratios indicate lower risk of attack (events: *n* = 49, observations: *n* = 180).

We first tested the hypothesis that the timing of rituximab administration (first‐line versus escalation) impacts the efficacy of attack suppression. Over their entire disease course (from the start of the first immunotherapy to the last follow‐up), the risk of attack was lower in individuals who received rituximab as first‐line therapy compared to escalation therapy (HR = 0.45, 95% CI = 0.30–0.67, *p* = 8.73 × 10^−5^, Figure 1A). However, there was no difference in the risk of attack after the start of rituximab (either administered as first‐line or as escalation therapy, HR = 1.15, 95% CI = 0.64–2.08, *p* = 0.64, mixed‐effects Cox proportional hazards model after time‐to‐event analysis with recurring events, patient as random effect, H_0_: HR = 1, Figure 1B). Next, we tested the association of clinical variables with the risk of attacks during rituximab treatment (administered as first‐line and as escalation therapy). Sex, age at diagnosis, and disease manifestation had no influence on the risk of attack (mixed‐effects multivariable Cox proportional hazards model, Figure 2). Patients receiving rituximab as escalation therapy had a longer time between disease onset and treatment, longer observation times, and more attacks before treatment (3 vs. 1 attack, Table 2). Age at diagnosis, age at start of rituximab, proportion of females, and phenotype at disease manifestation did not differ.

**TABLE 2:** Clinical characteristics of AQP4‐IgG+ NMOSD patients who received rituximab as first‐line or escalation immunotherapy.

	Rituximab first‐line	Rituximab escalation	*p*
Number of patients	52	81	—
Time between first manifestation and start therapy, median years (IQR)	0.38 (0.10–1.74)	4.59 (2.00–9.35)	4.10 x 10^‐10^
Observation time, median years (IQR)	5.98 (4.0–9.0)	9.58 (5.24–12.33)	1.00 x 10^‐3^
Age at diagnosis, median years (IQR)	48.5 (34.5–59.75)	46.0 (35.0–53.75)	0.29
Number of attacks before therapy start, median (IQR)	1 (1–3)	3 (2–6)	3.84 x 10^‐7^
Age at start therapy, median years (IQR)	49.18 (34.06–59.28)	49.03 (37.44–56.83)	0.92
Females, proportion	0.85	0.89	0.65
Myelitis/Optic neuritis/Other phenotype at onset, proportion	0.52/0.27/0.21	0.41/0.41/0.19	0.26

*Note:* *p* values result from chi‐squared test (proportion of females, proportion of disease phenotype at onset) or Mann–Whitney *U* test (all other variables) for the comparison between patients who received rituximab as first‐line therapy and patients who received rituximab as escalation therapy (IQR, interquartile range).

### 3.3 EDSS Worsening

The median EDSS score at the start of rituximab was higher in patients who received rituximab as escalation treatment (Md = 6.0, IQR = 3.5–6.5) compared to patients who received rituximab as first‐line treatment (Md = 3.0, IQR = 1.5–4.0, *p* = 1.10 × 10^−3^, Mann–Whitney *U* test, escalation: *n* = 38 patients, first‐line: *n* = 18 patients, Table 3). Sufficient (baseline and follow‐up) EDSS scores to assess EDSS score changes during the whole disease course were documented for 92 patients (first‐line rituximab immunotherapy: *n* = 34, rituximab as escalation immunotherapy: *n* = 58). Overall, 22 patients (24%) showed EDSS worsening over their entire disease course. Disease worsening was less common in patients who received rituximab as a first‐line treatment (*n* = 2/34, 6%) than in those who received it as an escalation immunotherapy (*n* = 20/58, *p* = 2.0 × 10^−3^, Fisher exact test, Table 3).

**TABLE 3:** Median EDSS scores at start of rituximab and EDSS worsening over the entire disease course in AQP4‐IgG+ NMOSD patients treated with rituximab.

	All	Rituximab first‐line	Rituximab escalation	*p*
EDSS score at start rituximab, median (IQR), *n*	4.0 (3.0–6.0), 56	3.0 (1.5–4.0), 18	6.0 (3.5–6.5), 38	1.10 × 10^−3^
EDSS score at last follow‐up/discontinuation of rituximab, median (IQR), *n*	4.0 (2.0–6.0), 92	2.75 (1.5–3.5), 34	5.5 (3.0–6.5), 58	2.31 × 10^−4^
Proportion of subjects with EDSS worsening	22/92 (24%)	2/34 (6%)	20/58 (34%)	2.0 × 10^−3^

*Note:* *p* values result from Mann–Whitney *U* test (EDSS scores at start rituximab, EDSS scores at last follow‐up/discontinuation of rituximab) or Fisher exact test (proportion of subjects with EDSS worsening; IQR, interquartile range).

### 3.4 Risk of Attacks Under Azathioprine and Mycophenolate Mofetil

**FIGURE 3:**
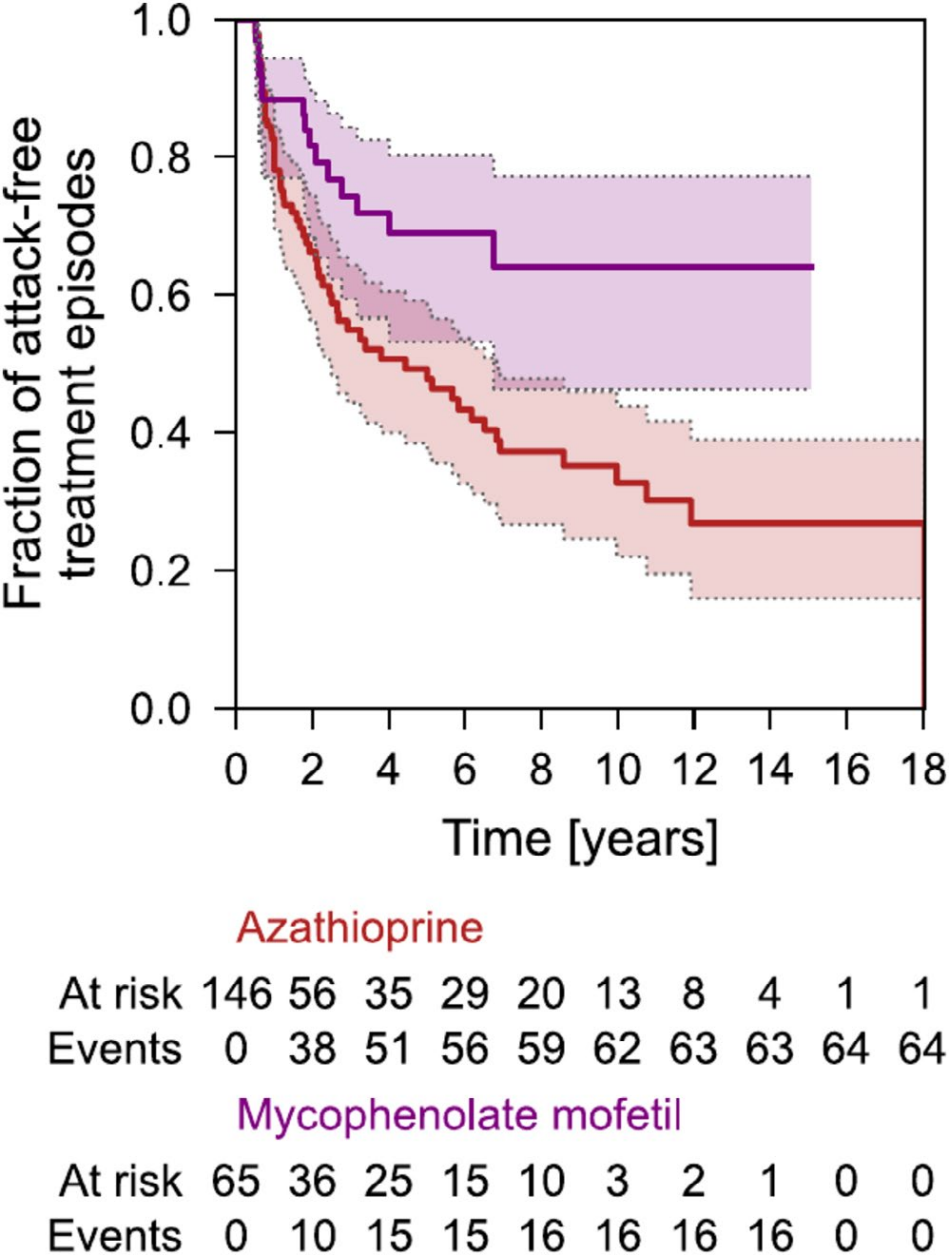
Risk of attacks. Time‐to‐event (attack) analysis for recurrent events in AQP4‐IgG+ NMOSD patients treated with azathioprine or mycophenolate mofetil.

**FIGURE 4:**
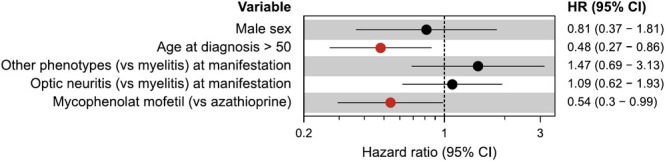
Risk factors for attacks under azathioprine and mycophenolate mofetil. Multivariable mixed‐effects Cox proportional hazards model based on recurring time‐to‐event analysis with patient as random effect. Dots represent hazard ratios, horizontal lines 95% confidence intervals. Lower hazard ratios indicate lower risk of attack (events: *n* = 80 observations: *n* = 211). Hazard ratios for variables with a 95% confidence interval excluding 1 are highlighted in red.

116 AQP4‐IgG+ NMOSD patients received azathioprine or mycophenolate mofetil as first‐line treatment. While most patients escalated to rituximab (*n* = 71), a subgroup (*n* = 45) continued treatment with azathioprine or mycophenolate mofetil. This subgroup had a lower risk of attack compared to those who initially received azathioprine or mycophenolate mofetil before escalating to rituximab (HR = 0.16, 95% CI = 0.08–0.29, *p* = 2.43 × 10^−9^). We next analyzed whether the overall risk of attack is different for azathioprine compared to mycophenolate mofetil. We observed a lower risk of attack in patients treated with mycophenolate mofetil compared to those treated with azathioprine (HR = 0.48, 95% CI = 0.27–0.86, *p* = 0.01, Figure 3). To identify factors associated with risk for attack under azathioprine and mycophenolate mofetil, we used a multivariable Cox proportional hazards model. Patients older than 50 years and patients treated with mycophenolate mofetil (in comparison to azathioprine) had a lower risk of attack (Figure 4). Sex or phenotype at disease manifestation (optic neuritis vs. myelitis) did not alter the risk of attack for subjects treated with conventional immunosuppression.

## 4 Discussion

In this study, we compared the risk of attack and disability worsening in AQP4‐IgG+ NMOSD patients receiving rituximab as first‐line or escalation treatment in a German and United Kingdom cohort. Overall, rituximab was highly effective in preventing attacks as both a first‐line and escalation immunotherapy, independent of age and sex. Disability worsening was more frequently observed in patients who received rituximab as escalation therapy (in contrast to first‐line), and individuals with rituximab as escalation immunotherapy showed higher EDSS scores at the start of rituximab, suggesting that an induction therapeutic strategy is superior to an escalation treatment approach.

Despite the approval of new therapies, conventional immunosuppressants and rituximab are still frequently used to suppress attacks in AQP4‐IgG+ NMOSD [20]. The choice of treatment is driven not only by individual medical needs but also by socioeconomic factors. In Germany, off‐label therapy with rituximab has been largely adopted as standard first‐line treatment (reimbursement following application to and approval by health insurers) during the past decade [21]. In contrast, in the United Kingdom, rituximab is only reimbursed as second‐line therapy, and most patients receive azathioprine or mycophenolate mofetil as first‐line treatment, which is well reflected in this study. These differences allowed us to directly compare first‐line and escalation treatment with rituximab in AQP4‐IgG+ NMOSD. Importantly, we found no differences in the clinical profile (e.g., onset location of attack) between German and United Kingdom subcohorts.

More than half of the patients were switched from conventional first‐line immunosuppression to rituximab, along with a lower risk of attacks in patients receiving rituximab in the first line. Thus, treating with highly effective therapies at the earliest timepoint is superior for attack suppression in AQP4‐IgG+ NMOSD patients. Furthermore, prior studies have shown that rituximab is superior to conventional immunosuppression in reducing attack rates [15, 22, 23, 24]. In AQP4‐IgG+ NMOSD, attacks drive disability [2, 25]. We hypothesized that patients with restricted access to rituximab (primarily treated first‐line with conventional immunosuppressants) due to healthcare system differences would have a worse outcome and accumulate more disability. Overall, the proportion of disability worsening during rituximab treatment episodes was low. However, fewer individuals treated first‐line with rituximab showed disability worsening when compared to those who received rituximab as escalation therapy (after failure of conventional immunosuppression). In line with this finding, a previous multicenter study from Korea observed an association between early rituximab treatment and disability prevention, especially in female patients with middle‐age onset and with severe attacks [16]. Similar results were observed in a Brazilian NMOSD cohort: the proportion of individuals with highest EDSS scores (6.5–9.5) decreased in a subcohort of patients who received rituximab early compared to those who received rituximab as escalation therapy [26]. Considering the effective attack suppression with rituximab both as first‐line and escalation therapy, the low rate of EDSS worsening with the use of rituximab as escalation treatment, and the higher EDSS scores at therapy start in patients receiving rituximab as escalation therapy, it can be assumed that disability accumulation primarily occurred as a result of incomplete attack remission from attacks which occurred under conventional immunosuppression.

We also identified individuals who received and continued either azathioprine or mycophenolate mofetil without escalating to rituximab. In a multivariable model, among the tested clinical and demographic parameters, we identified (older) age and treatment with mycophenolate mofetil (compared to azathioprine) predicting a favorable response to conventional immunosuppression. The effect of age is supported by a previous study which showed that a higher preceding disease activity and (younger) age could predict a worse response to conventional immunosuppression [27]. A greater reduction in attack rate with mycophenolate (compared to azathioprine) has already been reported in a previous study [28]. Previous systematic reviews and meta‐analyses highlighted the efficacy of mycophenolate mofetil in NMOSD but also demonstrated better tolerability compared to azathioprine [29, 30]. Whether a subgroup of patients with AQP4‐IgG+ NMOSD exhibits per se a milder disease course or responds particularly well to conventional immunosuppression such as mycophenolate mofetil remains unclear and needs further investigations. Recent data suggest that NMOSD with a benign disease course is rare [31]. Since most patients receiving ongoing conventional immunosuppression were from the United Kingdom, selection bias is an unlikely explanation for this observation. In the United Kingdom, therapy initiation with conventional immunosuppression largely occurs regardless of risk factors or disease severity, as reflected in our data on the distribution of first‐line immunotherapies.

In this retrospective analysis, no randomization was performed. However, the prospect of randomized controlled trials of unlicensed conventional immunosuppressants against licensed monoclonal antibodies is unlikely. The decision of when to use rituximab depended on individual factors such as disease activity but also the availability and feasibility of therapy. Nevertheless, the German and United Kingdom subcohorts and those receiving rituximab as first‐line or escalation treatment were comparable. Furthermore, the differences in the United Kingdom and German healthcare systems allowed us to compare rituximab as first‐line or escalation treatment usage. Regardless of the possible lower risk of EDSS worsening in patients who receive rituximab as first‐line immunotherapy, we were not able to assess other important relapse sequelae, such as quality of life and pain, which are known to worsen after NMOSD attacks. Another limitation of our study is the lack of detailed dosage information for azathioprine and mycophenolate mofetil. Since individual drug efficacy can be influenced by the administered dose, the absence of this data may represent a potential confounder when interpreting treatment outcomes in the conventional immunosuppression group. The strength of this study is that the data are derived from well‐characterized cohorts with regular follow‐ups. These results reflect real‐world use, benefits, and limitations of rituximab and conventional immunosuppression as disease‐modifying therapies in AQP4‐IgG+ NMOSD.

Our data underscore that first‐line rituximab should be prioritized in AQP4‐IgG+ NMOSD and can reduce disability accumulation. The availability of rituximab biosimilars may reduce costs and enhance treatment accessibility in resource‐constrained settings, bolstering the argument for B cell depletion as a preferred approach to treating AQP4‐IgG+ NMOSD. However, a subgroup of patients with AQP4‐IgG+ NMOSD may still respond well to conventional immunosuppression—specifically older patients treated with mycophenolate mofetil.

## Author Contributions

**Daniel Engels:** conceptualization, methodology, software, data curation, investigation, validation, formal analysis, supervision, project administration, resources, visualization, writing – original draft, writing – review and editing. **Joachim Havla:** investigation, data curation, resources. **Orhan Aktas:** investigation, data curation, resources. **Corinna Trebst:** investigation, data curation, resources. **Saif Huda:** conceptualization, investigation, data curation, supervision, resources, writing – review and editing, project administration, validation, methodology. **Tania Kümpfel:** writing – original draft, writing – review and editing, conceptualization, methodology, supervision, resources, data curation, project administration, validation, investigation. **Pakeeran Siriratnam:** investigation, data curation, resources. **Mirasol Forcadela:** investigation, data curation, resources. **Chiara Rocchi:** investigation, data curation, resources. **Marius Ringelstein:** investigation, data curation, resources. **Achim Berthele:** investigation, data curation, resources, writing – review and editing. **Katrin Giglhuber:** investigation, resources, data curation. **Martin W. Hümmert:** investigation, data curation, resources.

## Ethics Statement

The study was approved by the local ethics committees and all participants provided written informed consent.

## Conflicts of Interest

Daniel Engels received speaker honoraria and/or travel reimbursement from Alexion, Horizon/Amgen, Merck, and Roche, none related to this work. Chiara Rocchi, Mirasol Forcadela have nothing to disclose. Pakeeran Siriratnam has received travel support from Novartis and Biogen. Joachim Havla reports a grant for OCT research from the Friedrich‐Baur‐Stiftung, Horizon, and Merck; personal fees and nonfinancial support from Alexion, Amgen, Bayer, Biogen, BMS, Merck, Novartis, and Roche; and nonfinancial support from the Sumaira‐Foundation and Guthy‐Jackson Charitable Foundation, all outside the submitted work. Martin W. Hümmert received institutional research support from Myelitis e. V., German Federal Joint Committee/Innovation Fund, and NEMOS e. V. Speaker honoraria from selpers og, AMGEN/Horizon, and Alexion, travel grants from Alexion, and compensation for serving on an advisory board from Alexion, Roche, and UCB. None of this interfered with the current manuscript. Marius Ringelstein received speaker honoraria from Novartis, Bayer Vital GmbH, Roche, Alexion, Horizon, and Ipsen and travel reimbursement from Bayer Schering, Biogen Idec, Merz, Genzyme, Teva, Roche, Horizon, Alexion, and Merck, none related to this study. Katrin Giglhuber received reimbursement of traveling expenses from UCB. Achim Berthele receives funding from the Innovationsausschuss of the German Federal Joint Committee (G‐BA; grant 01VSF23040) and from the German Federal Ministry of Education and Research (BMBF; grant 01ZZ2102B). He has received consulting and/or speaker fees from Alexion, Argenx, Biogen, Horizon, Merck, Novartis, Roche, and Sandoz/Hexal, and his institution has received compensation for clinical trials from Alexion, Biogen, Merck, Novartis, Roche, and Sanofi Genzyme. Corinna Trebst received honoraria for consultation and expert testimony from Alexion Pharma Germany GmbH. None of this interfered with the current study. Orhan Aktas reports grants from the German Ministry of Education and Research (BMBF) and the German Research Foundation (DFG); grants and personal fees from Biogen and Novartis; and travel support and personal fees from Alexion, Almirall, MedImmune, Merck Serono, Roche, Sanofi, Viela Bio/Horizon Therapeutics, and Zambon. Saif Huda receives funding from an NIHR SCRPA Grant. Tania Kümpfel has received speaker honoraria and/or personal fees for advisory boards from Novartis Pharma, Roche Pharma, Alexion/Astra Zeneca, Horizon Therapeutics/Amgen, Merck, Chugai Pharma, and Biogen. The institution she works for has received compensation for serving as a member of a steering committee from Roche. She is a site principal investigator in several randomized clinical trials, and her institution has received compensation for clinical trials from Novartis Pharma, Roche Pharma, and Sanofi Genzyme; all outside the present work.

## Supporting information


**Table S1.**Demography of patients treated with azathioprine and mycophenolate mofetil before escalating to rituximab and ongoing. *p* values result from chi‐squared test (proportion of females, proportion of disease phenotype at onset) or Mann–Whitney *U* test (all other variables; IQR, interquartile range).

## Data Availability

The datasets generated and/or analyzed during the current study are not publicly available due to local regulations concerning the protection of patient data but are available from the corresponding author on reasonable request.
