# Supplementary material for: Rituximab as First‐Line Compared to Escalation Immunotherapy Is Associated With Lower Disability Accumulation in Aquaporin‐4‐IgG‐Positive Neuromyelitis Optica Spectrum Disorder: A Multicenter Cohort Study From Germany and the United Kingdom
Source: Eur J Neurol. 2025 Jun 10;32(6):e70243. doi: 10.1111/ene.70243 (PMC12150144; doi:10.1111/ene.70243)
Supplement: Supplementary file 1 — Table S1.Demography of patients treated with azathioprine and mycophenolate mofetil before escalating to rituximab and ongoing. p values result from chi‐squared test (proportion of females, proportion of disease phenotype at onset) or Mann–Whitney U test (all other variables; IQR, interquartile range). [file ENE-32-e70243-s001.docx]

|  | **Azathioprine/mycophenolate mofetil** | **Azathioprine/mycophenolate mofetil ongoing** | **Azathioprine/mycophenolate before escalating to rituximab** | **P value (Azathioprine/mycophenolate before escalating to rituximab vs Azathioprine/mycophenolate mofetil ongoing)** |
| --- | --- | --- | --- | --- |
| **Number of patients** | 116 | 45 | 71 | --- |
| **Time between first manifestation and start therapy, median years (IQR)** | 0.23 (0.0 - 0.58) | 0.25 (0.03 - 0.58) | 0.19 (0.0 - 0.63) | 0.83 |
| **Observation time, median years (IQR)** | 9.19 (5.64 - 13.01) | 9.2 (5.96 - 14.1) | 9.19 (5.22 - 12.13) | 0.33 |
| **Age at diagnosis, median years (IQR)** | 48 (38 - 57) | 53.5 (44.0 - 60.25) | 46.0 (32 - 55) | 0.005 |
| **Number of attacks before therapy, median (IQR)** | 1 (1 - 2) | 1.0 (1.0 - 2.0) | 1.5 (1 - 3) | 0.91 |
| **Age at start therapy, median years (IQR)** | 46.97 (38.12 - 57.78) | 53.91 (44.0 - 60.95) | 42.04 (31.31 - 54.44) | 0.001 |
| **Females, proportion** | 0.85 | 0.8 | 0.89 | 0.30 |
| **Myelitis/optic neuritis/other phenotype at onset, proportion** | 0.48/0.39/0.14 | 0.56/0.36/0.09 | 0.44/0.39/0.17 | 0.33 |

**Supplement Table 1.** Demography of patients treated with azathioprine and mycophenolate mofetil before escalating to rituximab and ongoing. P values result from chi-squared test (proportion of females, proportion of disease phenotype at onset) or Mann-Whitney U test (all other variables; IQR: interquartile range).
